# Emergency Treatment of Burns in Adults—Characteristics of Adult Patients and Acute/Pre-Hospital Burn Management

**DOI:** 10.3390/ebj6020019

**Published:** 2025-04-10

**Authors:** Bogdan Oprita, Georgeta Burlacu, Vlad Mircea Ispas, Ioana Adriana Serban, Ruxandra Oprita

**Affiliations:** 1 Emergency Department, Clinical Emergency Hospital of Bucharest, 105402 Bucharest, Romania; bogdan.oprita@umfcd.ro (B.O.); georgetaflorea77@yahoo.com (G.B.); vlad.ispas.mail@gmail.com (V.M.I.); 2 Faculty of Medicine, University of Medicine and Pharmacy “Carol Davila”, 050474 Bucharest, Romania; ruxandra.oprita@umfcd.ro; 3 Faculty of General Nursing, Bioterra University, 013722 Bucharest, Romania; 4 Gastroenterology Department, Clinical Emergency Hospital of Bucharest, 105402 Bucharest, Romania

**Keywords:** burns, first aid, emergency department, injury, burn management

## Abstract

Background: Burns represent one of the most severe injuries encountered in the pre-hospital and ED environment, with essential features and an often negatively powerful impact on patients’ quality of life. Preventive measures can significantly reduce the number of cases presenting to medical facilities; knowledge and the correct application of first aid measures in the pre-hospital stage have a significant role in reducing the risk of complications and in obtaining optimal outcomes. Methods: This retrospective one-year single-center study analyzed 399 adult burn patients treated at the Clinical Emergency Hospital of Bucharest (CEHB) in 2023. Information concerning the main characteristics of the patients (age, sex, and residence), etiology and severity of burns, and pre-hospital management of patients was analyzed. Results: Most patients (63.41%) resided in urban areas, with a higher prevalence of males (55.89%). Thermal burns accounted for 77.69% of cases, primarily caused by water, food, oil, or flames. Burns covered ≤10% TBSA in 77.19% of cases, while 6.52% extended beyond 50% TBSA. First aid was provided to 52.63% of patients at the accident site, often by non-specialized individuals. The mean time to presentation was 34.90 h, with significant correlations between time, age, burned body surface area, and burn depth. Conclusions: There is a real need for improvements in first-aid training and health initiatives to enhance pre-hospital burn care. Better documentation of the care provided to patients before being admitted to specialized centers, as well as further studies in this field, are absolutely necessary for improving prevention programs and burn management in the acute stage.

## 1. Introduction

The incidence and epidemiology of various medical conditions give a broad picture of the impact on individuals and society. Although most burns are not severe and can be treated on an outpatient basis [1], globally the latest estimates from the World Health Organization show that there are around 180,000 deaths each year are due to burn accidents [2]. Beyond that, however, the number of individuals (children and adults) who suffer such accidents is difficult to estimate, even approximately [3]. In Romania, there are no comprehensive data available on the incidence of burns or regarding burn management, especially on-site and in pre-hospital settings [4]. A welcomed initiative appeared only in April 2023 when the Single National Registry of Burn Patients was opened.

Burns are a very particular type of trauma in that they can lead to, in severe cases, significant changes in tissue proteins, localized edema, fluid loss, immunological and metabolic changes, systemic complications, systemic inflammatory syndrome, multiple organ failure, and exitus [5,6]. Beyond these, however, no less important are the psychosocial and emotional disturbances experienced by many patients [7].

Medical practice has shown that the evolution and the prognosis of the burned wound, i.e., of the burn patient, always depends on several cumulative factors that should never be ignored or underestimated: the patient’s age, the thermal agent and its temperature, the duration of action, the burn localization, the degree of depth, the affected body surface, the associated trauma, pre-existing chronic pathologies, the patient’s mental state, and the surgical treatment [8]. The etiologic agent determines the specific characteristics of the burn injury, and knowledge of the etiology and mechanism of the burn lesion, as well as its temperature and duration of exposure, provide important landmarks in deciding on the treatment strategy. Also, the etiologic agent imprints specific characteristics of burn lesions and, at the same time, is associated with specific concomitant trauma. A favorable outcome is mainly seen in superficial burns. Still, these can worsen even in the absence of the first medical act (first aid) or due to the application of empiric treatments [9].

Treatment of burns is complex and requires a multidisciplinary approach. The indications in national and international guidelines differ in many aspects, but all emphasize the importance of immediate care and a management strategy in the first 24 h [10,11,12,13,14]. Numerous studies argue that giving proper first aid significantly reduces morbidity and mortality [15,16,17]. However, while hospital burn therapy is constantly being studied, evaluated, and improved, there is a significant lack of information on first aid and pre-hospital care practices [18].

This study analyzes the characteristics of patients with burns and the care they received in the pre-hospital stage. It provides data on the epidemiology of burns in adults and information on the knowledge and application of first aid measures by both the general population and healthcare professionals.

## 2. Materials and Methods

For the present study, 399 patients treated for burn injuries at the Clinical Emergency Hospital of Bucharest (CEHB) from January to December 2023 were screened. It is important to note that Bucharest has three hospitals with burn centers. The CEHB is one of them, and it has six beds for critical burns. CEHB serves southern Romania and also receives patients from the rest of the country when there is no possibility of admitting patients to nearby burn centers. The data used for the statistical analysis were extracted from existing documents in the hospital archive, considering demographic variables (age, sex, and residence environment), variables relevant to the severity of the injuries (etiology, age, localization, surface and depth, presence of upper airway burns, general condition at admission and their evolution until discharge, etc.) as well as existing information on first aid measures provided in the pre-hospital stage, namely at the accident scene and during transport to the medical unit. Microsoft Excel 365 quantitative and qualitative analysis methods were used to analyze the data. The correlation coefficient r (Pearson) and the *p* coefficient (ANOVA test) with *n* − 2 degrees of freedom and a *p* < 0.05 relevance threshold were used to establish the interdependence and correlation between the different variables.

## 3. Results

### 3.1. Demography

**Figure 1:**
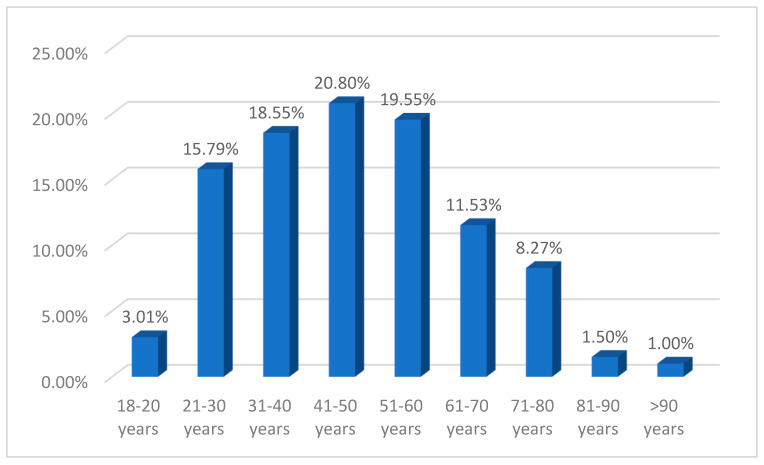
Distribution of patients by age groups.

Most patients (63.41%, *n* = 253) resided in urban areas, and there was also a relative prevalence of male patients (55.89%, *n* = 223). The mean age in this cohort was 46.9 ± 14 years (range 18–95 years), with relatively lower mean ages in male patients (45.7 ± 13.7 years vs. 48.6 ± 14.4 years in females) and in those with urban residence (45.9 ± 13.9 years vs. 48.9 ± 13.8 years for those with rural residence). The highest number of patients was found in working-age patients, i.e., those aged between 21 and 65 years, who accounted for 80.95% (*n* = 323) of the total study group (Figure 1).

### 3.2. Etiology

**Figure 2:**
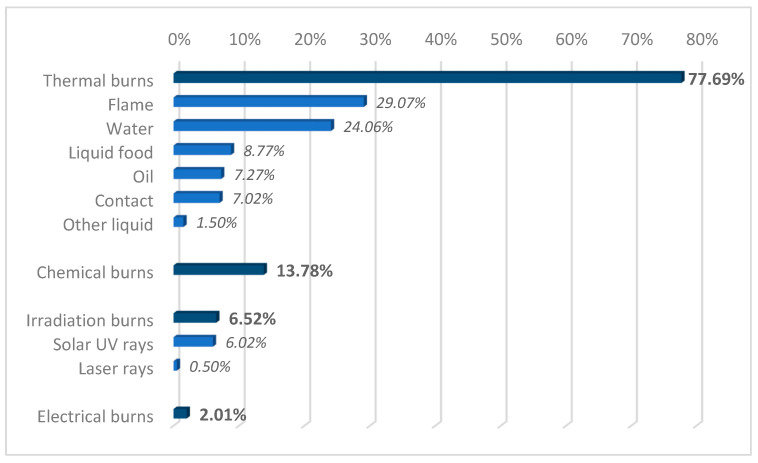
Etiology of burn injuries.

In terms of etiology, thermal burns (77.69%, *n* = 310) accounted for the largest share, and most of them were caused by water, food, or oil (40.10%, *n* = 160), or flame (29.07% of all cases, *n* = 116). A significant percentage of thermal flame burns were caused by explosions (58 cases, 14.54% of the total). The chemical burns were caused by contact with corrosive substances (acids, chlorine, antifreeze, gasoline, herbicides, insecticides, paints, cement, and cleaning products) or cosmetics, and radiation burns were acquired because of overexposure to the sun (24 cases, 6.52%) (Figure 2).

**Figure 3:**
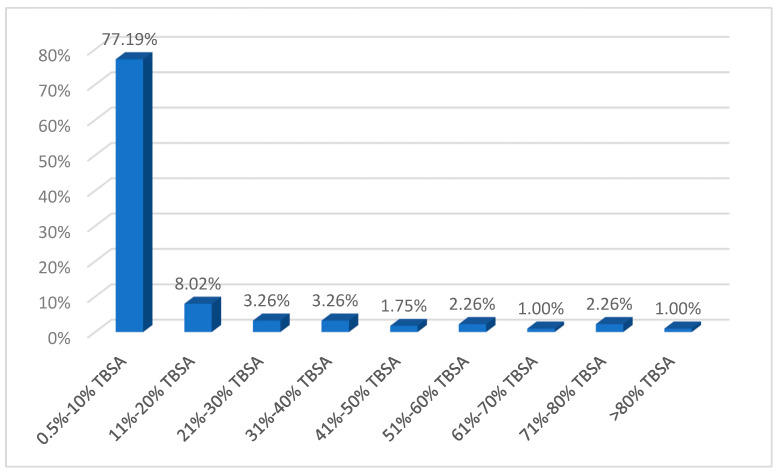
Extension of burn lesions.

In 77.19% of cases (*n* = 308), burn injuries covered ≤10% TBSA, and the proportion of patients presenting with burns extending more than 50% TBSA was 6.52% (*n* = 26) (Figure 3). Considering the etiology of burns, electrocution injuries had on average the highest extent of coverage (31 ± 27% TBSA). Of the thermal burns, flame burns were associated with the most extensive post-combustion injuries (mean 26.37 ± 22.22% TBSA). It is also worth mentioning that in the case of self-ignition and blast accidents, patients also suffered extensive injuries (77.50 ± 17.50% TBSA and 33.17 ± 22.74% TBSA, respectively).

**Figure 4:**
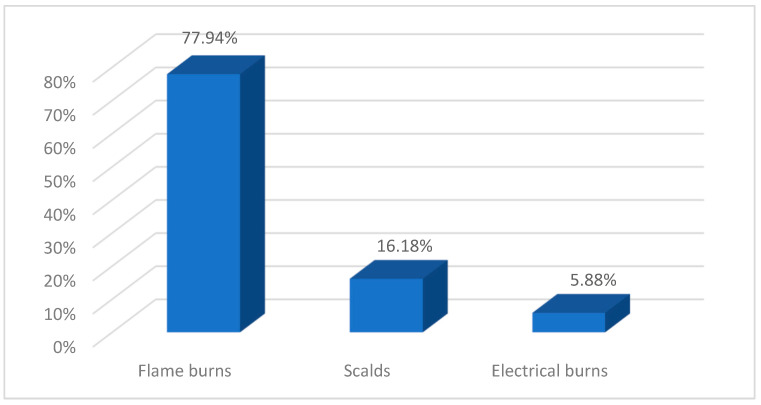
Etiology of severe burns.

Out of all patients in the study, 17.04% (*n* = 68) had extensive lesions while having a TBSA ≥20%. From a demographic perspective, this group of patients has a higher average age than those with less-extensive lesions (52.5 ± 13.9 years vs. 45.83 ± 13.7 years) and a significantly higher percentage of male patients (82.35% vs. 17.04%). Thermal burns, particularly those caused by flame, are the most common etiology (Figure 4). In 48.53% (*n* = 33) of cases with extensive burns, these occurred in the context of explosions.

**Figure 5:**
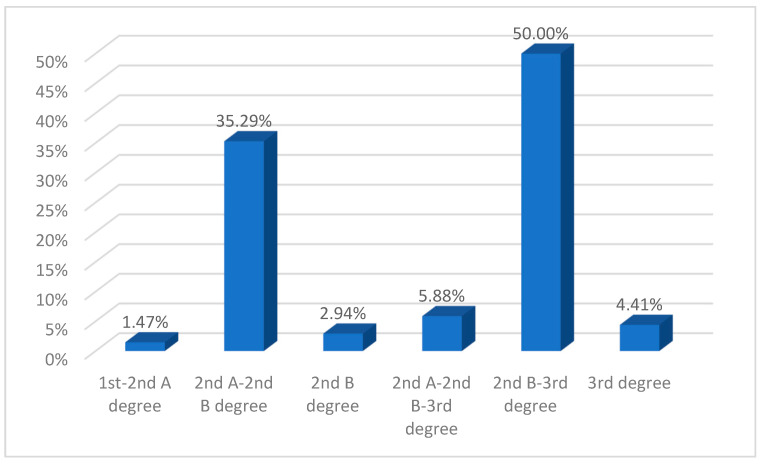
Depth of extended burn injuries (≥20% TBSA) (percentages calculated based on the number of patients with extended burn injuries, *n* = 68).

Most patients included in the present study presented first- and second-degree burns (83.21% of cases, *n* = 332: Table 1). It was found that in patients with third-degree burns, the lesions had a significantly higher mean lesion extension of 34.22 ± 24.53% TBSA compared to a mean of 6.51 ± 6.85% TBSA in patients with first- and second-degree burns. As for extensive lesions (≥20% TBSA), they were also associated with greater burn depth (Figure 5).

Upper airway burns were found in 49 patients (12.28% of the total), with these patients presenting with significantly extensive (48.08 ± 21.22% TBSA on average) and deep (grades 2nd B and 3rd) lesions. Most patients in this category suffered thermal flame burns in the context of explosions.

Associated trauma was diagnosed in 11 cases (2.76% of the total), with a predilection for contusions, head trauma, fractures, and cardio-respiratory arrest. These traumas were associated with thermal burns (flame/explosion or hot liquids) or electrocution.

From the perspective of the severity of the burn injuries, there were found some differences between male and female patients, and between those residing in urban and, respectively, urban areas, in the rural area. However, it should be noted that these differences are based on descriptive observations, as no statistical analysis was conducted to validate their significance. Both men and patients living in rural areas presented more severe injuries, with higher proportions of extensive burns ≥20% TBSA, deep burns, and more cases of airway burns and associated trauma (Table 2).

### 3.3. Time to Presentation

**Figure 6:**
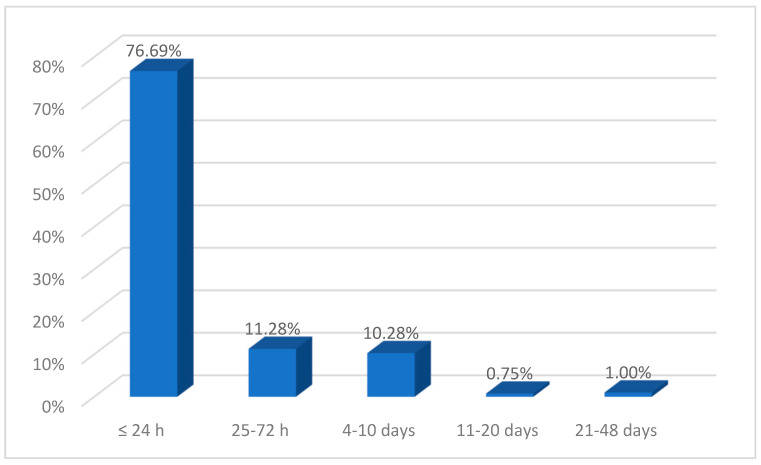
Time to presentation in burn lesions.

The mean time to presentation at the emergency room was 34.90 ± 43.70 h, with most patients (76.69%, *n* = 306) receiving treatment in the CEHB within 24 h of receiving their burns (Figure 6). Data analysis revealed significant correlations between the time to presentation, patient age, burned body surface area, and burn depth (Table 3). It was also found that female patients were brought to the emergency room at significantly higher mean intervals compared to males (41.85 ± 48.57 h vs. 29.42 ± 38.69 h) as well as to rural patients (39.62 ± 51.35 h vs. 26.73 ± 29.83 h for urban). Another observation is that patients with airway burns and those with associated trauma arrived at the hospital in a considerably shorter time interval after the burn occurred (12.14 ± 13.29 h and 5.55 ± 6.71 h, respectively) compared to those without airway burns (39.09 ± 47.09 h) or associated trauma (35.73 ± 44.54 h).

### 3.4. Pre-Hospital Care

Of the 399 patients included in this study, 210 (52.63%) received first aid at the accident site, as recorded in the medical records. Disaggregating the group according to this criterion, it was observed that the main characteristics differentiating the two categories were those related to the severity of the burns (those who received first aid had burns over a significantly larger body surface area and deeper injuries: both airway burns and associated trauma were more frequent).

A significant percentage of patients were transported from the accident scene by their own means, and it is evident that first aid was provided by non-specialized people, mainly using water, various creams or yogurt, oil, honey, etc. In cases where an ambulance was called, first aid was provided on-site following the protocols in force, applying specialized burn dressings and then performing interventions according to the general condition of the patient and the severity of the burn injuries and associated trauma (Table 4).

## 4. Discussion

Most studies addressed issues related to certain categories of patients, especially those hospitalized in specialized burn units or patients with certain types of burns. Therefore, we have selected two similar studies to conduct a relevant discussion on the incidence of burns in adults. For Romania, we identified a single study that included all burn cases (355 patients) presented at one of the largest emergency centers in the northeast of the country, namely „Sf. Spiridon” Hospital, from Iasi [19]. We chose the study conducted by Koucheck et al. in Iran, which involved a cohort of 2213 patients presented at Shahid Motahari Hospital in Tehran, as one of our international research selections [20].

The ratio of men to women was 1.27:1, which was in line with Pieptu et al. [19] or Kouchek et al. (1.54:1) [20] and international estimates indicating a prevalence of male patients [21]. The mean age of the patients is also close to the previous study in Romania (44.3 years), but significantly higher than in the Iranian study (34.98 years) [19,20].

Epidemiological conclusions regarding the rural–urban ratio should be drawn with caution. Urban areas are the most common location for patients in most studies reviewed [4,19,20], particularly when discussing less severe burns. Therefore, it can be considered that many of the patients with such lesions presented themselves and were treated in non-specialized centers or did not resort to medical services.

Hot liquids caused the majority of burns, which aligns with epidemiological findings worldwide and in previous reports [17,19,20,21]. The high incidence of explosion-related burns in this study is notable and may be attributed to workplace accidents or household incidents involving liquefied petroleum gas cylinders or improvised heating installations. These types of injuries are often severe and require immediate medical attention [22].

As can be seen from the results of the present study, severe burns (in terms of surface area and depth) are rarer, and their incidence has decreased in recent years, especially in developed countries [21]. This study aimed to conduct a comprehensive analysis of all cases in which patients presented themselves to the emergency department of CEHB. The results of the statistical analysis demonstrated a notably high proportion of patients with burns covering ≤10% TBSA (77.19% of the total, *n* = 308), as well as a 6.02% incidence (*n* = 24) of first-degree burns. These findings are consistent with those reported by Pieptu et al., who identified an 85.19% prevalence of patients presenting with burns covering <10% TBSA [19].

With regard to the cases of first-degree burns, the following observations should be noted, that the majority (11 cases) were solar burns, 8 patients sustained thermal burns (from contact and scalding), and 5 experienced chemical burns. Twenty-three of the twenty-four patients presented to the emergency department independently and all received medical treatment, but none met the criteria for admission to the burn center. Consequently, it is inferred that the decision of patients to present to the emergency department is influenced by personal choice, a lack of information, or mistrust regarding treatment options in non-specialized medical facilities. Additionally, it may be attributed to a lack of awareness regarding first aid measures and the appropriate care for minor burns.

Second-degree burns (diagnosed in most of the cases analyzed here—93.73%, *n* = 370) often show dynamic changes in the early post-burn period, changes that are determined not only by their pathophysiologic characteristics but also by the existence of trauma and other factors. Timely and reasonable pre-hospital first aid and appropriate treatment of wounds after hospitalization are essential to prevent wound deepening. However, there are still many variations in the treatment of deep second-degree wounds in both the acute and post-acute phases (how to change the dressing, choice of external dressing or medications, indication for appropriateness, and timing of surgery) [23].

In a pre-hospital setting, medical professionals face multiple challenges in treating burn victims and examining patients for associated trauma; assessing the severity of burns in terms of both burned area and depth is often difficult [18]. The risk of complications increases exponentially when the call to the ambulance service or the transport of patients to the emergency room is delayed.

According to the protocol in force in Romania, patients with burns acquired in the context of explosions, those with burns to the face, with an altered voice, with burns to the nasal wings, etc., are suspected to have an airway injury [24,25]. To protect the airway, these patients are intubated, and the burns below the glottis are subsequently classified in the burns ward. Consequently, in this study, any involvement of the airways was categorized as an airway injury, without differentiating between supraglottic and subglottic burns or between airway burns and injuries resulting from smoke or carbon monoxide inhalation. After the first aid given by ambulance crews, management decisions belong to the clinicians who assess and treat the patients, and the approach to cases presupposes “clinical gestalt”—the attending physician’s decision based on clinical experience, multidisciplinary assessment, and functional assessment. In cases where subglottic injuries (severe trauma with dynamic progression) are suspected, treatment decisions are grounded in an interdisciplinary approach. The execution of complex and invasive diagnostic procedures, such as fiberoptic bronchoscopy, necessitates consultations with specialists in otolaryngology, pulmonology, anesthesia, and plastic surgery and is carried out in the burn unit/center.

Three other important points to consider when discussing presentation time involve several factors. So far, we have not identified any relevant socio-cultural elements that explain why women tend to present later for specialized burns treatment. However, based on the statistical results, we can infer that this delay is likely due to a tendency to initially seek traditional remedies and non-specialized medical care or to overlook less severe burns. Socioeconomic factors such as geographical challenges and access to healthcare facilities, awareness of burn severity, and decision-making processes within households might also influence the delay in women seeking medical care. For instance, patients with injuries less than 20% TBSA presented an average of 44.45 h after the accident for women and 35.40 h for men, while those with injuries of 20% TBSA or more reached the CEHB in 6.25 h for women and 11.59 h for men.

Additionally, it is important to note that 13.53% (*n* = 54) of the patients included in the study group, particularly those from rural areas, were initially treated at other medical centers before being transferred to CEHB, and the time to presentation for these patients was 181.78 h (compared to 11.91 h for patients brought directly from the accident site). Therefore, the presentation time recorded in the statistical data reflects the duration of these initial admissions. From this perspective, it is important to note that, in the group of patients with <20% TBSA, the proportion of women initially treated in other hospitals was higher than that of men (17.68% vs. 13.17%), leading to a longer mean presentation time for women. In the subgroup of patients with ≥20% TBSA, 5.36% of male patients were transferred from other hospitals, whereas all female patients were directly admitted to the burn center from the accident site, resulting in a longer mean presentation time for men. In cases of severe burns, airway damage, and/or associated trauma, the short time from the accident to presentation at the CEHB is primarily due to the use of air ambulances.

It is important to note that a significant percentage of patients in this study (47.37%, *n* = 189) did not receive first aid at the scene of the accident, and many of them presented to the hospital more than 24 h after the burns occurred, even though they were of a significant degree of severity. We believe that this demonstrates either a certain emotional incapacity of those who could have given first aid or a lack of education regarding the importance of first aid in the event of such trauma. On the other hand, there was a relatively low percentage (12.78%, *n* = 50) of cases in which various “folk remedies” (creams, honey, oil, yogurt, cabbage, etc.) were used. While many studies and guidelines argue for using water to cool burns [15], only 12.78% of cases were recorded, suggesting that non-specialized people and medical professionals only use the remedy to a small extent.

In the study by Wood et al., it was found that first aid was granted in particular in the case of scalding and burning by flame [15]. In our study, first aid interventions were reported for 87.50% of patients with electrocutions, for 73.28% of patients with flame burns, 70.91% of those with chemical burns, and only 34.94% of patients with scalding. However, it should be specified that for electrocutions and flame burns ambulance service is required as a rule, while in other cases folk remedies are sometimes applied. It was also noted that patients who did not receive first aid had, on average, slightly enlarged lesions (3.68 ± 14.06% TBSA). First aid and presentation time at the hospital were not related to the depth of burn injuries.

There is still essential debate in the literature regarding the indicators for intubation of patients, and studies are arguing for avoiding intubation when it is unnecessary [26,27,28]. According to our protocol, oro-tracheal intubation should not be delayed if signs of respiratory distress are present or anticipated. Intubation before transport is considered prudent for patients to be transferred to specialized burn centers. Importantly, in the induction sequence, succinylcholine may be used for the first 72 h in patients with severe burns at the time of burns, but not thereafter as it may increase the risk of hyperkalemia [29].

Supplemental oxygen administration (100%) and airway protection are essential in the management of an inhalation injury. If mechanical ventilation is required, low tidal volumes are preferred to minimize airway pressure. Permissive hypercapnia (respiratory acidosis) may be used to maintain low pressures but should be avoided if there is concurrent head trauma [29,30].

There are also discussions regarding fluid administration, with some recommending overloading which may lead to complications [18,31]. A general agreement is, however, expressed in terms of stopping the burning process, cooling the burned areas, avoiding hypothermia and adequate analgesic administration, rational fluid administration, and ensuring airway function and circulation [11,18,32,33,34].

Medically assisted transport (by land or air) to a specialized center is indicated in the first hours after the burn, but only if the patient is hemodynamically and respiratory balanced. Otherwise, a complication such as an infection, hemorrhage, heart failure, or multiple organ failure may occur. In cases where patients present with such complications, transportation should be delayed or canceled. In the pre-hospital stage, during transport, the priority is to maintain vital functions in normal parameters, and this may involve changes in fluid therapy, type of mechanical ventilation, fitting of venous lines, or administration of analgesics [35,36,37].

One of this study’s key limitations is its reliance on self-reported data for pre-hospital interventions. The accuracy of these reports may be influenced by recall bias, inconsistencies in documentation, and variations in first-responder training. Additionally, the lack of standardized reporting protocols may lead to either an over- or under-estimation of first aid measures applied at the scene. Future research should consider incorporating direct observations, structured interviews, or the digital tracking of pre-hospital care to enhance data reliability and minimize these biases.

## 5. Conclusions

The administration of first aid on-site and the care of burn patients in a pre-hospital setting is extremely important for avoiding complications and achieving optimal healing outcomes for patients thereafter.

Evidence demonstrates the need for regular studies analyzing patient characteristics and treatment strategies in pre-hospital settings, which provide important insights into the epidemiology of burn injuries, the interventions needed in terms of preventive measures, education of the population and healthcare providers, and solutions to improve clinical practice. Our findings highlight the need for targeted improvements in first-aid training and public health initiatives to enhance pre-hospital burn care. We propose the following key recommendations: expand first-aid education programs in schools and workplaces, focusing specifically on burn management and emergency response; strengthen training for emergency responders, particularly regarding airway burns and criteria for early intubation; launch public awareness campaigns to promote evidence-based first aid measures and discourage harmful traditional remedies (e.g., use of oils, yogurt, or toothpaste on burns); develop and implement national guidelines for standardized pre-hospital burn care, ensuring consistency in treatment protocols and decision-making regarding transport to specialized centers.

Future research should further investigate the differences between patients who receive first aid and those who do not. This approach would benefit the revision of protocols used by healthcare professionals and public education interventions.

## Figures and Tables

**Table 1:** Depth of the burn injuries.

	2nd A–2nd B Degree	3rd Degree
0.5–10% TBSA	66.92% (*n* = 267)	4.26% (*n* = 17)
11–20% TBSA	4.26% (*n* = 17)	3.76% (*n* = 15)
21–30% TBSA	1.75% (*n* = 7)	1.50% (*n* = 6)
31–40% TBSA	2.01% (*n* = 8)	1.25% (*n* = 5)
41–50% TBSA	0.50% (*n* = 2)	1.25% (*n* = 5)
51–60% TBSA	1.25% (*n* = 5)	1.00% (*n* = 4)
61–70% TBSA	-	1.00% (*n* = 4)
71–80% TBSA	0.25% (*n* = 1)	2.01% (*n* = 8)
>80% TBSA	-	1.00% (*n* = 4)

**Table 2:** Burn severity in men vs. women, patients with urban residence vs. rural residence.

	Men	Women	Rural	Urban
1st degree	5.83% (*n* = 13)	6.25% (*n* = 11)	3.42% (*n* = 5)	7.51% (*n* = 19)
2nd A-B degree	73.99% (*n* = 165)	81.25% (*n* = 143)	70.55% (*n* = 103)	81.03% (*n* = 205)
3rd degree	20.18% (*n* = 45)	12.50% (22)	26.03% (*n* = 38)	11.46% (*n* = 29)
TBSA ≥ 20%	25.11% (*n* = 56)	6.82% (*n* = 12)	30.14% (*n* = 44)	9.49% (*n* = 24)
Airway burns	17.04% (*n* = 38)	6.25% (*n* = 11)	21.23% (*n* = 31)	7.11% (*n* = 18)
Associated trauma	3.14% (*n* = 7)	2.27% (*n* = 4)	4.79% (*n* = 7)	1.58% (*n* = 4)

**Table 3:** Correlations among the mean delay from injury to visiting the burn center and age, burnt body surface, and the depth of the burn injury.

Variable	R	*p*	Trust
Age	0.87	0.0025	99%
TBSA%	−0.61	0.0025	99%
Depth of the burn injury	−0.84	0.0025	99%

**Table 4:** Patient characteristics depending on the provision of first aid at the place of the accident.

	Patients with First Aid at the Place of the Accident %(*n*) *	Patients Without First Aid at the Place of the Accident %(*n*) *
Sex		
Masculine	63.81% (134)	47.09% (89)
Feminine	36.19% (76)	52.91% (100)
Residential environment		
Urban	54.76 (115)	73.02% (138)
Rural	45.24 (95)	26.98% (51)
Average age		
Total	48.08 ± 14 years	49.07 ± 13.88 years
Men	44.63 ± 13.70 years	47.22 ± 13.57 years
Women	45.87 ± 14.52 years	50.71 ± 14.06 years
Burnt body surface (mean %TBSA)	18.50 ± 14.06%	3.68 ± 14.06%
Burn degree		
1st degree	5.71% (12)	6.35% (12)
1st–2nd A/2nd B degree	14.76% (31)	21.69% (41)
2nd A–2nd B degree	54.76% (115)	64.02% (121)
2nd–3rd degree	24.76% (52)	7.94% (15)
Mean delay from injury to burn center	17.20 ± 14.06% h	34.90 ± 14.06% h
Inhalation injury	22.38% (47)	1.06% (2)
Associated injuries	3.81% (8)	1.59% (3)
Taken from the place of the accident		
Own means	47.14% (99)	97.35% (184)
Ambulance	21.90% (46)	2.65% (5)
PA Ambulance	5.24% (11)	-
UTIM Ambulance	5.71% (12)	-
Plane ambulance	2.38% (5)	-
Helicopter ambulance	17.62% (37)	-
Pre-hospital interventions		
Oro-tracheal intubation	22.86% (48)	1.06% (2)
Peripheral venous line	40.48% (85)	2.65% (5)
Central venous catheter	19.05% (40)	1.59% (3)
Urinary catheter	22.38% (47)	1.59% (3)
Analgesia	44.29% (93)	2.12% (4)
Parkland formula	34.76% (73)	2.12% (4)
Patients coming from other medical unit	23.81% (50)	2.12% (4)
Hospitalization		
Yes	41.90% (88)	20.11% (38)
In Bucharest Clinical Emergency Hospital	38.57% (81)	19.58%
In other mnedical units	3.33% (7)	0.53% (1)
No	58.10% (122)	79.89% (151)
Did not need hospitalization	55.71% (117)	77.78% (147)
Refused hospitalization	2.38% (5)	2.12% (4)

* The calculation of the percentage weights was performed by referring to the number of patients in every category. TBSA—total body surface area; EPA ambulance—first aid team ambulance; UTIM ambulance—mobile intensive care unit ambulance.

## Data Availability

The original data presented in the study are included in the article, further inquiries can be directed to the corresponding author.
